# Supracardiac Total Anomalous Pulmonary Venous Return With Extrinsic Compression of the Vertical Vein by the Left Pulmonary Artery: A Case Report

**DOI:** 10.1155/crpe/5873152

**Published:** 2026-09-15

**Authors:** Abdolla Alnaqshi, Idriss Wakaa Al Ahmad, Bishr Al-abdulrazzak, Hanady nabel zwaraa, Lorieh Edmon Domat, Jean Domat, Mouhammed Sleiay, Mohammad Younes

**Affiliations:** ^1^ Faculty of Medicine, Damascus University, Damascus, Syria, damascusuniversity.edu.sy; ^2^ Cardiology Department, Damascus Hospital for Heart Diseases and Surgery, Damascus, Syria; ^3^ Pediatric Surgery Department, Children University Hospital, Damascus University, Damascus, Syria, damascusuniversity.edu.sy; ^4^ Plastic Surgery Department, Almouwasat University Hospital, Damascus University, Damascus, Syria, damascusuniversity.edu.sy; ^5^ Faculty of Medicine, Al-Mouwasat University Hospital, Damascus University, Damascus, Syria, damascusuniversity.edu.sy; ^6^ Faculty of Medicine, Hama University, Hama, Syria; ^7^ Department of Pediatric Cardiac Surgery, Faculty of Medicine, Damascus University, Damascus, Syria, damascusuniversity.edu.sy

**Keywords:** congenital heart defect, echocardiography, postoperative complications, total anomalous pulmonary venous return (TAPVR), vertical vein compression

## Abstract

**Background:**

Supracardiac total anomalous pulmonary venous return (TAPVR) is a rare congenital heart defect. An anatomical variant where the vertical vein courses posterior to the left pulmonary artery, causing extrinsic compression and severe pulmonary venous obstruction, is exceptionally uncommon and life‐threatening.

**Case Presentation:**

We describe the case of a male infant aged 2.5 months who exhibited severe central cyanosis, respiratory distress, and right heart failure. In the absence of computed tomography (CT), diagnosis was based exclusively on 2D echocardiography, which effectively detected obstructed supracardiac TAPVR with a vertical vein compressed behind the left pulmonary artery, as well as a secundum atrial septal defect (ASD). A small pulmonary venous confluence was discovered intraoperatively. A customized surgical method was used, making use of the cut vertical vein to enhance a broad anastomosis between the confluence and the left atrium. The postoperative course was complicated by severe respiratory failure and neurological events, such as a generalized seizure accompanied by a 3‐min asystole and subsequent periventricular leukomalacia. Even with these serious challenges, thorough medical management resulted in complete recovery. At the 7‐month follow‐up, the patient demonstrated stability, with unobstructed pulmonary venous flow and normalized cardiac geometry.

**Conclusion:**

In settings with limited resources, echocardiography alone can provide an accurate diagnosis of atypical and severely obstructed TAPVR. Even in the presence of serious neurological and respiratory complications during the perioperative phase, it is possible to obtain excellent long‐term results by means of adaptive surgical methods aimed at widening a small venous confluence and thorough postoperative intensive care.

## 1. Introduction

Total anomalous pulmonary venous return (TAPVR) is a congenital heart defect characterized by the anomalous connection of all pulmonary veins, which drain into the systemic venous circulation rather than the left atrium (LA), resulting in a left‐to‐right shunt and frequently cyanosis. This malformation accounts for approximately 1% to 3% of all congenital cardiac anomalies, with an incidence rate ranging from 0.6 to 1.2 per 10,000 live births (1, 2, 3 4).

Anatomically, TAPVR is classified into four main subtypes based on the site of anomalous drainage. The supracardiac type is the most common variant, representing 45% to 55% of cases, followed by the cardiac type (20%–25%), the infracardiac type (11%–25%), and the mixed type, which is the least frequent (5%) [1, 2]. The infracardiac type is most susceptible to pulmonary venous obstruction (occurring in over 90% of cases), leading to respiratory distress and high mortality [2].

In recent decades, advances in surgical techniques and perioperative management have markedly improved patient outcomes. Surgical correction involves rerouting the anomalous pulmonary veins to the LA and is typically undertaken during the neonatal period. Current data indicate that early postoperative mortality rates are now less than 10% [1]. Accurate preoperative morphological characterization, traditionally achieved through echocardiography remains the primary diagnostic modality, though cardiac catheterization and angiography continue to serve as the gold standard for definitive diagnosis [3], is essential to mitigate the risk of postoperative complications, particularly pulmonary venous stenosis [2]. Furthermore, mutations in genes of the retinoic acid pathway (such as retinol binding protein 5 [RBP5] and NODAL) and other previously implicated genes (including PDGFRA and ANKRD1) have been associated with genetic susceptibility to TAPVR [4].

It is noteworthy that fewer than 7% of untreated patients survive into adulthood, with survival typically associated with the supracardiac type and the absence of pulmonary venous obstruction [3].

We describe a rare and severe case of supracardiac TAPVR in an infant, characterized by an atypical pattern of pulmonary venous drainage in the presence of an atrial septal defect (ASD), complicated by significant compression of the vertical vein.

## 2. Case Presentation

**FIGURE 1:**
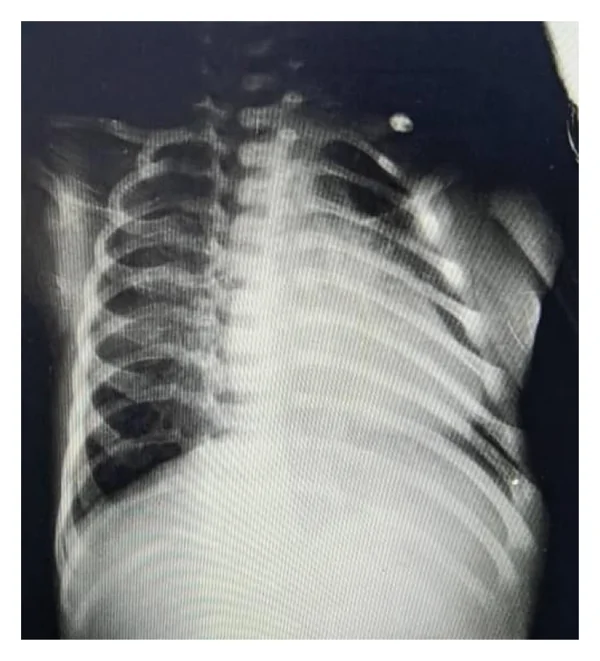
Chest radiography demonstrating mild bilateral pulmonary infiltrates.

A 2.5‐month‐old male infant, weighing 4.5 kg, presented with moderate dyspnea, intercostal and subcostal retractions, and central cyanosis exacerbated by crying. Oxygen saturation was 60% on room air. Physical examination revealed fine crackles and mild expiratory wheezing, a 2/4 systolic murmur, a palpable but nonvisible apex beat, and mild hepatomegaly (2 cm below the costal margin). The anterior fontanelle was open and bulging. Chest radiography demonstrated mild bilateral pulmonary infiltrates (Figure 1).

Echocardiography confirmed supracardiac TAPVR with elevated pulmonary pressures, a vertical vein coursing posterior to the left pulmonary artery, and a secundum ASD. Doppler assessment demonstrated accelerated pulmonary venous flow with a peak velocity of approximately 1.6 m/s and an estimated mean gradient of 6–8 mmHg across the pulmonary venous confluence, consistent with significant preobstructive physiology. Although computed tomography (CT) imaging was not performed due to unavailability, the echocardiographic findings were considered sufficient for anatomical delineation and surgical planning.

The vertical vein was visualized only at its junction with the innominate vein; the distal segment was not fully visualized preoperatively due to its posterior course behind the left pulmonary artery and acoustic shadowing from overlying vascular structures, which is a recognized limitation in supracardiac TAPVR imaging.

Following median sternotomy and initiation of cardiopulmonary bypass, the vertical vein was visualized only at its junction with the innominate vein, which appeared normal and noncongested. In contrast to typical obstructed supracardiac TAPVR—where the innominate vein is usually dilated, tortuous, and exhibits evidence of elevated venous pressure with sluggish or turbulent flow—our case demonstrated preserved caliber and laminar flow, suggesting a more distal site of obstruction.

**FIGURE 2:**
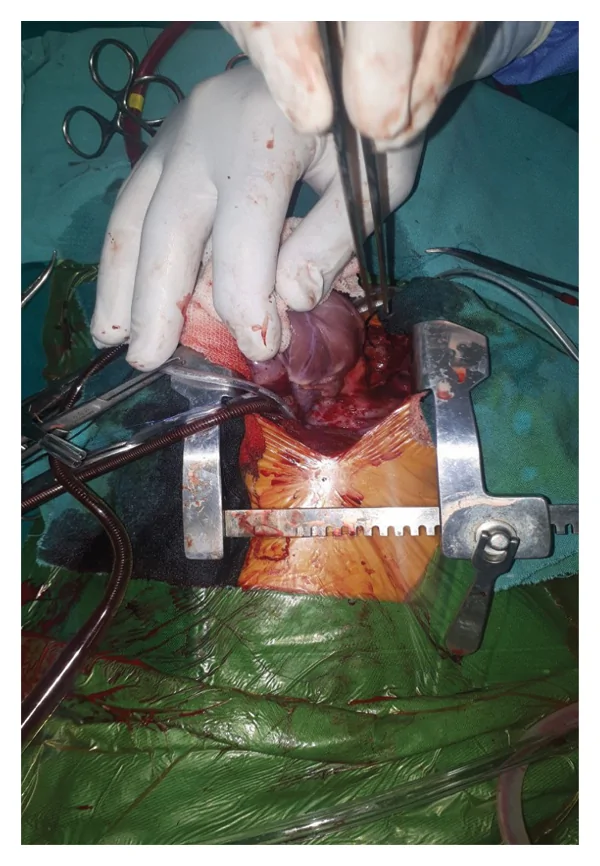
Dissection of the pulmonary artery and its left branch revealing the vertical vein passing posterior to the left pulmonary artery, causing extrinsic compression.

Dissection of the pulmonary artery and its left branch revealed the vertical vein passing posterior to the left pulmonary artery, causing extrinsic compression (Figure 2). Preoperative echocardiography had already suggested a fixed point of narrowing at the level of the left pulmonary artery crossing, and this was subsequently confirmed intraoperatively as the true site of obstruction.

The venous confluence was small and mildly tense. Intraoperative inspection confirmed increased wall tension of the confluence with reduced collapsibility, and Doppler echocardiography correlated this finding with elevated inflow velocities, supporting the presence of functional obstruction rather than a purely descriptive anatomical observation.

The vertical vein was transected at its junction with the innominate vein and mobilized from surrounding structures. A sutureless repair technique (in situ pericardial marsupialization of the pulmonary venous confluence to the LA) was performed to minimize the risk of postoperative anastomotic stenosis, given the small size of the confluence and the presence of preoperative pulmonary venous obstruction.

The venous confluence was incised along the entire length between the right and left pulmonary veins, and the incision was extended through the vertical vein to its distal transected end. The previous incision was anastomosed to the posterior aspect of the LA.

The secundum ASD was closed using a relatively large bovine pericardial patch to enlarge the LA, leaving a 4‐mm central fenestration. Intraoperative decision for fenestration was made to allow controlled decompression of the LA in the setting of elevated pulmonary vascular resistance.

Temporary quadripolar pacing wires were placed on the right atrium and ventricle due to transient sinus bradycardia.

Postoperatively, the patient required moderate inotropic support.

On Postoperative Day 2, echocardiography revealed moderately reduced left ventricular systolic function with preserved right ventricular function and unobstructed laminar flow across the pulmonary venous anastomosis, with a mean Doppler gradient of < 2 mmHg and no evidence of residual stenosis.

Gradual weaning of inotropes was initiated. On Day 3, extubation failed due to severe respiratory distress, necessitating reintubation. On Day 5, the patient experienced a generalized tonic‐clonic seizure with a 3‐min period of asystole, which was managed successfully with resuscitation. Brain ultrasound via the fontanelles demonstrated mild ventricular enlargement and periventricular leukomalacia, likely secondary to hypoxic‐ischemic injury.

Subsequent extubation attempts on Day 11 failed, but the patient was successfully extubated on Day 16. He was transferred from the cardiac intensive care unit on Day 24 and discharged home after two months in stable condition.

Follow‐up at seven months postoperatively demonstrated unobstructed pulmonary venous drainage into the LA, with laminar flow across the pulmonary venous pathway and no evidence of recurrent stenosis (mean Doppler gradient < 2 mmHg). Cardiac chamber size and geometry had normalized. The atrial septal fenestration remained patent, measuring approximately 3‐4 mm, with a small low‐velocity left‐to‐right shunt on color Doppler and no evidence of hemodynamic compromise.

## 3. Discussion

TAPVR is a rare cyanotic congenital heart defect characterized by the abnormal drainage of all pulmonary veins into the systemic venous circulation or the right atrium, rather than the LA. This anomaly results from the persistence of embryonic connections between the pulmonary and cardinal veins during development [1–5].

Anatomically, TAPVR is classified into four main types based on the site of venous drainage. The supracardiac type is the most prevalent, representing 45% to 55% of cases, followed by the cardiac type (20% to 25%), the infracardiac type (11% to 25%), and the mixed type (5%) [1, 2, 6–9]. In our case, the type is supracardiac associated with ASD.

Historically, the infracardiac variant has been associated with a poor prognosis due to a high frequency of venous obstruction, with mortality rates exceeding 90% in untreated cases [8]. However, early postoperative mortality has significantly declined to less than 10% in specialized centers, with a mean age at repair of approximately one month [1].

Fewer than 7% of patients with TAPVR survive to adulthood. This rare outcome is typically observed in the supracardiac subtype where no pulmonary venous obstruction is present [3].

Regarding risk factors, pulmonary venous obstruction is a well‐established predictor of mortality, particularly in infracardiac TAPVR, where the vertical vein is prone to extrinsic compression [2]. Recent evidence, however, challenges the traditional view of the cardiac type as a benign variant. A large cohort study by Karamlou et al. demonstrated a significant association between cardiac‐type TAPVR and adverse outcomes, including mortality and the need for reintervention. This highlights the necessity for precise anatomical characterization prior to surgical planning. Anatomical risk factors such as a small pulmonary venous confluence orifice and an irregular drainage pattern into the right atrium have been implicated in the development of postoperative pulmonary vein stenosis (PPVS) [6]. Additionally, genetic factors have gained attention, with the PLXND1 gene identified as a significant contributor, accounting for approximately 4.17% of cases and demonstrating a high odds ratio for disease association [7]. These findings underscore the multifactorial nature of TAPVR and emphasize the importance of integrating anatomical, clinical, and genetic risk factors in the preoperative assessment and long‐term management of affected patients.

The clinical presentation of TAPVR is highly heterogeneous, depending primarily on the pathological anatomy, the association with other cardiac anomalies, and the degree of vascular obstruction. Typical presentation of TAPVR usually composed of cyanosis, signs of right heart volume overload as pulmonary blood flow increases, and moreover, other cardiorespiratory signs and symptoms. In a cohort study of 34 TAPVR patients, the predominant symptoms were dyspnea and cyanosis [8]. While infants with obstructed TAPVR are critically ill soon after birth presenting with cyanosis and signs of respiratory distress [9] requiring urgent surgical repair [10], infants with nonobstructed TAPVR are often asymptomatic at birth, but within the first year of life they present with tachypnea, poor feeding, or other symptoms and signs related to respiratory distress [9].

Our patient’s presentation at 2.5 months of age highlights a subacute but severe form of obstruction. Since all pulmonary venous blood returns to the right atrium, the ASD is the only pathway for blood to reach the left heart and the systemic circulation, but the presence of vertical vein compression restricts the total volume of blood reaching the heart, making the adequacy of the ASD shunting even more critical for maintaining systemic output resulting mild hepatomegaly and the bulging anterior fontanelle, which is indicative of severe systemic venous hypertension. In our patient, the observed cyanosis and profound dyspnea, crackles and wheezing were direct consequences of pulmonary venous hypertension caused by the extrinsic compression of the vertical vein leading to pulmonary congestion and, thus, difficult work of breathing.

The imaging findings vary according to the subtype of TAPVR. The first examinations to be conducted are chest radiography and echocardiography [10–13]. While the classic “snowman” sign on chest radiography is a hallmark of supracardiac TAPVR, it can be absent in other subtypes of TAPVR [14, 15]. Consequently, TAPVR diagnosis relies primarily on echocardiography and CT angiography. A retrospective study demonstrated that 2D echocardiography with color Doppler successfully diagnosed all 36 TAPVR cases studied, with cardiac catheterization and MRI serving as confirmatory tools [16]. On the other hand, a comparative study compared these modalities in 107 pediatric patients, finding that CT angiography was more reliable than echocardiography for detecting obstructive and mixed TAPVR types (66.4% vs. 83.2% nonobstructive detection), while both modalities performed similarly for supracardiac TAPVR detection (53.3% vs. 47.7%) [17].

In the case of our patient, echocardiography confirmed supracardiac TAPVR with elevated pulmonary pressures, a vertical vein coursing posterior to the left pulmonary artery and a secundum ASD. CT was not performed to our patient due to unavailability. Even so, echocardiography served to rule out other causes of neonatal cyanosis and respiratory distress, such as primary lung disease or persistent pulmonary hypertension of the newborn (PPHN) allowing for the transition to emergent surgical correction.

The management of anomalous pulmonary venous return (APVR) constitutes a complex, multidisciplinary challenge, with strategies ranging from urgent neonatal surgery to expectant monitoring in adulthood. The therapeutic approach is dictated by the anatomical subtype—TAPVR versus partial (PAPVR) anomalous pulmonary venous return—the presence and severity of symptoms, the resultant hemodynamic burden, and the association with other cardiac anomalies.

TAPVR is a critical congenital heart defect that is universally fatal without timely surgical intervention. Patients typically present symptomatically at birth with cyanosis and respiratory distress, necessitating early surgical repair [3].

The primary objectives of surgical correction are to create an unobstructed confluence between the pulmonary veins and the left atrium (LA), ligate the anomalous systemic venous connection, and close the associated ASD [1, 3].

Advances in surgical techniques and perioperative care have led to significant improvements in outcomes, with early mortality rates now less than 10% in contemporary series [1]. Survival into adulthood without surgical repair is exceptionally rare but has been documented, particularly in supracardiac variants without pulmonary venous obstruction and with a large, nonrestrictive ASD. Such an ASD allows for adequate mixing of pulmonary and systemic venous blood and decompression of the pulmonary veins, enabling long‐term survival. The oldest reported patient to undergo successful repair was 57 years of age [3].

In extreme cases with delayed presentation and a hypoplastic left heart, a staged surgical approach may be necessary. Planinc et al. (2022) describe a 7‐month‐old infant with a closed foramen ovale– and ductus‐dependent systemic circulation who was initially deemed inoperable. The team first performed a surgical atrial septectomy to create an adequate interatrial communication. This initial step promoted left heart growth, stabilized the patient hemodynamically, and allowed for a successful definitive TAPVR repair two months later [1].

While surgical repair remains the mainstay of treatment, ongoing research is elucidating the genetic etiology of APVR. These insights may eventually lead to novel therapeutic targets or improved risk stratification.

Utilizing shared segment analysis and whole‐genome sequencing, Nash et al. (2015) identified the retinoic acid signaling pathway as a key susceptibility factor in TAPVR. Their research found an overrepresentation of a deleterious variant in the RBP5 gene among TAPVR patients. Functional studies in zebrafish demonstrated that the orthologous gene, rbp7a, is critical for left‐right patterning. Furthermore, combined knockdown of rbp7a and the NODAL ortholog spaw resulted in laterality defects, suggesting a potential role for digenic inheritance in the pathogenesis of TAPVR [4].

## 4. Conclusion

This case demonstrates that unusual anatomical variations of supracardiac TAPVR, like the left pulmonary artery exerting external pressure on the vertical vein, can lead to critical pulmonary venous blockage that necessitates immediate surgery. Moreover, it highlights that in settings where resources are limited and advanced imaging techniques such as CT angiography are not available, comprehensive 2D echocardiography is a highly dependable and adequate method for making definitive diagnoses and planning surgeries. In the end, using adaptive surgical methods—such as utilizing the vertical vein to enhance a hypoplastic pulmonary venous confluence—along with thorough intensive care for serious postoperative complications, can result in outstanding long‐term clinical and hemodynamic outcomes.

## Funding

The authors have nothing to report.

## Disclosure

The work has been reported in line with the SCARE criteria [18].

## Ethics Statement

The authors have nothing to report.

## Consent

Written informed consent was obtained from the patient’s legal guardian for publication of this case report and accompanying images. A copy of the written consent is available for review by the Editor‐in‐Chief of this journal on request.

## Conflicts of Interest

The authors declare no conflicts of interest.

## Data Availability

Data sharing is not applicable to this article as no datasets were generated or analyzed during the current study.
